# Polycystic Ovary Syndrome: A Brain Disorder Characterized by Eating Problems Originating during Puberty and Adolescence

**DOI:** 10.3390/ijms21218211

**Published:** 2020-11-03

**Authors:** Régine P. M. Steegers-Theunissen, Rosalieke E. Wiegel, Pauline W. Jansen, Joop S. E. Laven, Kevin D. Sinclair

**Affiliations:** 1 Department of Obstetrics and Gynaecology, Erasmus MC, University Medical Centre, 3000 CA Rotterdam, The Netherlands; r.wiegel@erasmusmc.nl (R.E.W.); j.laven@erasmusmc.nl (J.S.E.L.); 2 Department of Child and Adolescent Psychiatry/Psychology, Erasmus MC, University Medical Centre, 3000 CB Rotterdam, The Netherlands; p.w.jansen@erasmusmc.nl; 3 Department of Psychology, Education and Child Studies, Erasmus University Rotterdam, 3000 DR Rotterdam, The Netherlands; 4 School of Biosciences, Sutton Bonnington Campus, University of Nottingham, Leicestershire LE12 6HD, UK

**Keywords:** PCOS, emotional disturbance, psychological stress, nutrition, eating disorders, DNA methylation, prevention, neuroendocrine hormones

## Abstract

Polycystic ovary syndrome (PCOS) is an endocrine condition associated with reproductive and psychiatric disorders, and with obesity. Eating disorders, such as bulimia and recurrent dieting, are also linked to PCOS. They can lead to the epigenetic dysregulation of the hypothalamic–pituitary–gonadal (HPG) axis, thereby impacting on ovarian folliculogenesis. We postulate that PCOS is induced by psychological distress and episodes of overeating and/or dieting during puberty and adolescence, when body dissatisfaction and emotional distress are often present. We propose that upregulated activation of the central HPG axis during this period can be epigenetically altered by psychological stressors and by bulimia/recurrent dieting, which are common during adolescence and which can lead to PCOS. This hypothesis is based on events that occur during a largely neglected stage of female reproductive development. To date, most research into the origins of PCOS has focused on the prenatal induction of this disorder, particularly in utero androgenization and the role of anti-Müllerian hormone. Establishing causality in our peripubertal model requires prospective cohort studies from infancy. Mechanistic studies should consider the role of the gut microbiota in addition to the epigenetic regulation of (neuro) hormones. Finally, clinicians should consider the importance of underlying chronic psychological distress and eating disorders in PCOS.

## 1. Introduction

Polycystic ovary syndrome (PCOS) is the most common disorder in women during the reproductive period, accounting for around 80% of anovulatory subfertility [1]. Obesity, early features of cardiometabolic diseases, including insulin resistance and hyperinsulinemia, and a range of mental illnesses are also associated with PCOS [2,3,4,5]. The estimated prevalence of PCOS in women of reproductive age ranges between 9% and 18%, with the highest prevalence in Western countries [5,6,7]. Variations in these rates have mainly been attributed to the use of different diagnostic criteria. PCOS is, by definition, a normo-gonadotropic, normo-estrogenic state and is often associated with anovulation. PCOS is diagnosed according to the Rotterdam 2003 consensus with typical features including clinical or biochemical hyperandrogenism, irregular menstrual cycles and the so-called polycystic ovarian morphology [8]. The diagnostic criteria for PCOS, particularly during adolescence, are controversial because many features used in adult women, such as acne, irregular menses and polycystic ovary morphology (PCOM), can be normal physiological characteristics of puberty [9].

Although PCOS presents in numerous women during adolescence, coincident with pubertal activation of the hypothalamic–pituitary–gonadal (HPG) axis, it can affect women at any stage during the life course. Signs of precocious pubarche and adolescent hyperandrogenism with or without obesity, and insulin resistance may indicate the early stages of PCOS [10]. PCOS has long been considered a primary syndrome of ovarian dysfunction. However, recent evidence has started to shed light on neuroendocrine impairments associated with the pathophysiology of this syndrome [11]. In women with PCOS, the function of the gonadotropin-releasing hormone (GnRH) pulse generator is perturbed, giving rise to luteinizing hormone (LH) overproduction and a relative follicle-stimulating hormone (FSH) shortage. This over-secretion of LH further increases androgen production by the theca cells surrounding the follicle. Historically, this has been attributed to a lack of negative feedback from progesterone as a result of hypothalamic prenatal androgenization [11]. In women with PCOS, anti-Müllerian hormone (AMH) serum levels are also frequently found to be 2- to 3-fold higher than in women with healthy ovaries. Moreover, the severity of the PCOS phenotype correlates with AMH production, which is higher in anovulatory than in ovulatory PCOS patients [12].

**Figure 1:**
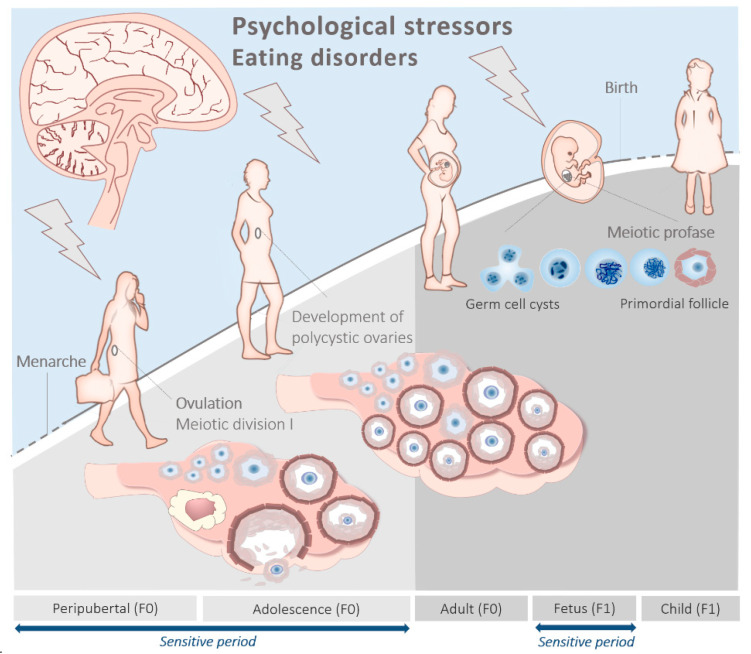
The impact of alternating recurrent periods of psychological stressors and eating disorders in the critical periods during the life course in the developmental origins of polycystic ovary syndrome (PCOS). Modified from Steegers et al.’s Textbook of Obstetrics and Gynaecology; a life course approach, 2019 [13].

Although overproduction of AMH is central to our understanding of the pathophysiology of PCOS, we propose a novel and complementary hypothesis that considers PCOS as an adverse psychological condition which develops in women at the onset of puberty and throughout adolescence as a consequence of stress, mood problems and low self-esteem, together with metabolic disturbances arising from eating disorders, which constitute a secondary confounder (Figure 1). Furthermore, we propose that intrauterine exposure to psychological distress and to the eating disorder pathology, in part mediated by AMH, can predispose offspring to PCOS. We further consider that this is particularly so in mothers who may themselves be polycystic, or who had endured periods of psychological stress perhaps leading to the onset of eating disorders during adolescence.

## 2. Linking Psychological Distress and Eating Disorders to PCOS

Women of reproductive age with PCOS are more often affected by mild to moderate psychiatric disorders, such as mood and eating disorders [2,14,15,16]. Whilst most studies investigating these associations have been of a cross-sectional design, one of the few longitudinal studies undertaken indicated that, among women with PCOS, around 20% developed a depressive disorder within two years [17]. Several underlying psychological and neuroendocrine pathways could explain this observation. Firstly, accompanying symptoms, such as menstrual irregularity and subfertility, can contribute to psychological distress, eventually resulting in a psychiatric disorder [8]. Similarly, changes in appearance like weight gain, acne and scalp hair thinning may contribute to alterations in body image and a diminished self-esteem. However, these physical symptoms do not seem to fully account for the emotional disturbances experienced by PCOS patients [18], indicating that other mechanisms are also involved.

Mood and eating disorders are among the most common psychiatric disorders observed in women with PCOS and most likely the result of complex interactions between biological, sociocultural, familial and individual factors. The proposed diathesis–stress model of psychopathology suggests that a (biological) vulnerability for a mental illness is only expressed when it is triggered by a certain degree of stress. Indeed, stress is an important trigger for both depression and eating disorders. In bulimia nervosa and binge eating disorder, binge eating may occur as a coping mechanism for stress, as it brings temporary relief. Moreover, stress has also been associated with poor self-esteem and body image dissatisfaction [19], core symptoms of mood and eating disorders. Stressors may also affect health and well-being through neuroendocrine impairments. Stressful events lead to the production of several hormones (cortisol, epinephrine and norepinephrine) associated with the fight-or-flight response. While historically essential to survive, chronic elevated levels of stress-related hormones are detrimental for health and potentially play a causal role in the derangement of neuroendocrine pathways, exacerbating the development of PCOS. Although the full etiology of most psychiatric disorders has yet to be elucidated, impairments of neuroendocrine pathways associated with PCOS are also common features of associated psychiatric disorders. In both depression and anxiety, for example, serotonin imbalances and high basal cortisol levels are often observed [20]. Elevated testosterone concentrations may also promote food cravings, perhaps via a poor impulse control [21], which provides an explanation for the link between eating disorders and PCOS. Finally, psychological stress may also interfere with serum AMH levels, as discussed later, illustrated by the high levels of stress that have been related to decreased levels of AMH in subfertile women [22].

Given that most studies on PCOS and psychiatric disorders were of a cross-sectional design, the direction of effect in this association remains to be determined [14,15]. Whilst Kerchner et al. [17] described new cases of depression after the onset of PCOS in their longitudinal study, it was unclear whether these cases already had high sub-clinical levels of depressive symptoms when PCOS was diagnosed. Although several plausible arguments suggest that PCOS contributes to the development of psychiatric disorders, both conditions may also be expressions of the same underlying pathways. Yet, the alternative hypothesis in which physiological stressors associated with psychiatric disorders cause PCOS is also likely.

### 2.1. Eating Disorders and Impaired Neuroendocrine Pathways in PCOS

Eating disorders disturb neuroendocrine pathways leading to altered biochemical processes. Recurrent binge eating can increase insulin levels, by decreasing concentrations of sex hormone-binding globulin that can increase free circulating testosterone [23], thereby negatively impacting follicular maturation and ovulation [24]. More generally, stress also increases insulin levels which contribute to the development of PCOS by similar routes [25]. Another pathway relates to the reward system in the brain, which is involved in the regulation of binge eating and food cravings. Craved and binged foods are usually high in fat and/or sugar, resulting in a satisfied feeling due to the activation of dopamine neurons in the reward pathway. Yet, increased energy intake also promotes weight gain and increases insulin levels. Elevated insulin levels in turn stimulate ovarian androgen production, thereby contributing to a vicious circle of androgens and obesity [26].

Activation of the hypothalamic–pituitary–adrenal axis in response to psychological stress results in continuous secretion of cortisol. In particular, binge eating is correlated with high cortisol levels [27]. As mentioned before, the primary trigger during binge eating is stress, which repeatedly activates the adrenal cortex for cortisol secretion. This consistent stimulation could explain the increase in cortisol levels observed in PCOS women [28].

Obesity, which is common in, but not exclusive to, binge eating disorders, can disrupt normal appetite signaling regulated by leptin and ghrelin, two important hormones controlling hunger and satiety. Diet-induced obesity in rodents, who were given high doses of leptin (an appetite suppressor), reduced food intake, but this effect lasted only around two weeks. It seems that the rodents developed hypothalamic leptin resistance [29], which can lead to leptin overproduction. Leptin also has a reproductive function, acting at many levels within the hypothalamic–pituitary–ovarian axis. In particular, it has been suggested that the leptin receptors in the ovary among some obese patients may be overexposed to leptin, which in turn may affect ovarian function [30].

The circulating gut hormone ghrelin (acylated ghrelin, AG) regulates appetite and increases food intake and adiposity [31]. However, ghrelin has also been shown to interact with the brain reward pathways as it promotes the rewarding aspect of a high-fat diet in rodents [32,33]. Rodents fed ad libitum developed robust binge eating behaviors when fed high-fat diets. This intermittent access to palatable food induces hyperphagia in satiated mice [34]. Importantly, mice lacking the ghrelin receptor Gshr1 are resistant to binge eating and consume less calories [35]. These findings highlight the crucial role of ghrelin signaling in the development of altered eating behavior and food preference. The natural occurring antagonist of ghrelin, unacylated ghrelin (UAG), inhibits ghrelin action [31] and the AG/UAG ratio in patients with hyperphagia is elevated [36]. Furthermore, treatment with UAG analogs prevents diet-induced obesity in mice [37]. Hence, UAG may be a new treatment option for PCOS to break the vicious circle of androgen excess and obesity.

In addition, reduced serotonin levels are reported in the serum of PCOS women [38], which inhibit the pulsatile release of GnRH/LH [39]. Eating disorders have been linked to genes that are involved in the regulation of the neurotransmitter serotonin [40], which modulates both appetite, mood and circadian rhythm. Research showed that patients with anorexia nervosa (AN) had low levels of 5-hydroxyindolacetic acid (5-HIAA), which is indicative of low serotonin levels. This may be the result of the restricted food intake, as serotonin is made from tryptophan which is an essential nutrient. However, after recovery, these women showed higher levels of 5-HIAA than healthy women. A suggested explanation for this somewhat counterintuitive finding is that AN is caused by abnormalities in the serotonin system, in particular by serotonin overactivity in different brain areas. Yet, a reversed mechanism may be at play as well, with AN causing an overdrive of the serotonin system, in response to the previous shortage of serotonin.

### 2.2. Eating Disorders, Gut Microbiota and Intermediary Metabolism

Although detailed consideration is beyond the scope of the current article, there is an increasing body of evidence to implicate the gut microbiota in the etiology and progression of eating disorders, together with a broad range of related psychiatric conditions, obesity and the establishment of metabolic disease (including insulin resistance) and PCOS [41,42,43]. The composition of the gut microbiome can influence the absorption of various metabolites, such as short-chain fatty acids, bile acids and amino acids, together with inflammatory mediators (e.g., Interleukin-22) [44], the aforementioned gut-brain peptides (e.g., ghrelin and glucagon-like peptide 1 [GLP1]) and various neuromodulators (e.g., γ-amino butyric acid [GABA]) involved in mood regulation (41). Each of these metabolites can independently or collectively act on neuroendocrine pathways to induce a state of insulin resistance and hyperandrogenism, which in turn can predispose to PCOS [45]. Indeed, a recent cross-sectional study of Chinese obese and non-obese women with PCOS revealed significant enrichment in microbiota with active one-carbon (1C) and closely related purine and pyrimidine metabolic pathways, as well as enhanced tricarboxylic acid and lipopolysaccharide metabolic activities [45]. Inhabiting a unique niche at the point of intestinal absorption, gut microbes exhibiting these features will have undoubtedly contributed to the altered metabolic and endocrine characteristics of PCOS women in these study populations.

Lam et al. [41] explained that food restriction and limited food choices, both associated with eating disorders and obesity, can modify the gut microbiota and generally lead to a reduction in diversity associated with poor clinical outcomes. Gut microbes are also responsive to maternal (eukaryotic) hormonal signals, including neurohormones (e.g., norepinephrine, serotonin) that can be modified during psychological stress [45].

### 2.3. Eating Disorders and Diet Composition

Nutritional status is very clearly implicated in the (patho) physiology and treatment of PCOS. Interestingly, androgens stimulate appetite, food craving and recurrent binge eating, although the underlying mechanisms are not understood [21,46,47]. In an explorative study, we observed that women with PCOS were frequently dieting, very often consuming substandard foodstuffs relative to that consumed by the control population [48]. These substandard diets were characterized by low serum folate and elevated homocysteine concentrations, indicating a more general derangement in 1C metabolism associated with a hyperandrogenic status in these women.

Moreover, positive associations were established between an inadequate diet and AMH, and the free androgen index in PCOS patients. This is consistent with recent findings from our periconception cohort demonstrating that strong adherence to a healthy dietary pattern is associated with the non-hyperandrogenic PCOS phenotype and lower plasma AMH concentrations [49]. Several other studies have found differences in dietary intake between PCOS patients and healthy controls [50,51]. In our recent study, we did not observe increased total energy intakes between hyperandrogenic (HA) and non-HA PCOS patients. This is in contrast to Moran et al. [52], who reported an overall increase in energy intake, with improved diet quality, in PCOS patients. Furthermore, PCOS women were more likely to consume a Mediterranean dietary pattern [53]. This finding could be the consequence of adopting a healthy dietary pattern after having been diagnosed with PCOS. Dietary interventions that, for example, involve intermittent periods of fasting [54] and/or the use of insulin sensitizers (e.g., inositol isoforms) [55], can lead to improvements of PCOS features, substantiating the hypothesis that diet can indeed affect neuroendocrine pathways that regulate metabolic and reproductive functions [50,56,57]. Yet, the composition of the ideal weight-loss diet leading to improved PCOS features, such as hyperandrogenism, cycle regularity and metabolic and psychiatric outcomes, remains to be elucidated, as study outcomes to date have been conflicting [51,58,59,60,61,62,63]. Such diets vary in the content of carbohydrates, proteins and fat, or some combination of these components. Therefore, in our opinion, weight loss should always be accomplished by adequate intakes of healthy foods.

## 3. The Peripubertal and Adolescent Origin of PCOS

The risk of developing an eating disorder increases around puberty onset and further rises through adolescence, mediated in part by increasing estrogen levels [64]. Considering the important role of estrogen in gene transcription, studies suggest that estrogen may be involved in the activation of some genetic factors that affect eating disorders [65]. We have investigated genetic differences between women with high or low estrogen levels during puberty, and found that high levels of estrogens were correlated with substantial genetic effects on disturbed eating [66]. In addition, retrospective studies have found that precocious puberty onset is related to the prevalence of eating disorders [67] and psychological stress [68].

### Peripubertal Metabolism, Stress and Eating Disorders

**Figure 2:**
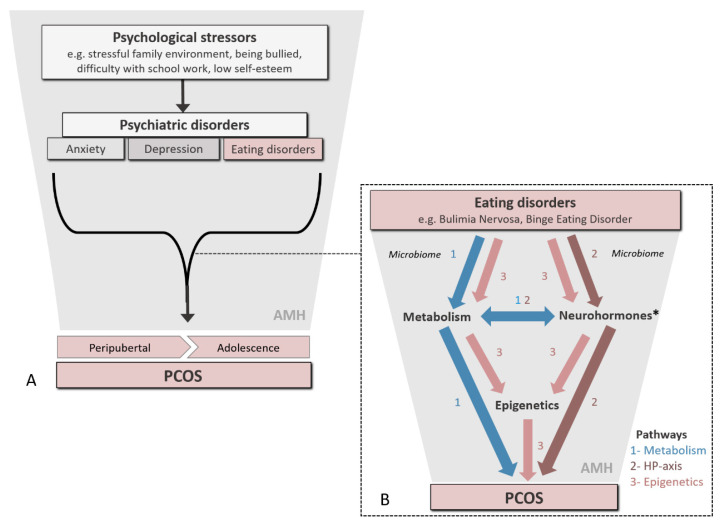
(**A**) Novel hypothesis for the role of psychological stressors and eating disorders in the induction of PCOS during the peripubertal and adolescent periods, emphasizing the importance of anti-Müllerian hormone (AMH) as an underlying factor. (**B**) Physiological pathways by which eating disorders during this critical period of development can contribute to the induction of PCOS. * Estradiol, AMH, leptin, insulin and ghrelin.

Childhood obesity and excessive nutrient intake are known to independently advance the onset of puberty in girls [69,70], leading to abnormal (neuro) endocrine activity during adolescence, which can potentially predispose to PCOS [71]. In general, high dietary intakes of energy, protein and polyunsaturated fatty acids are associated with early puberty onset, whereas high-fiber and monounsaturated fatty acid diets are associated with later menarche onset. Furthermore, the incidence of eating disorders in girls with PCOS is increased in overweight and obese subjects [72], which, in turn, may serve to further exacerbate the condition (Figure 2).

**Figure 3:**
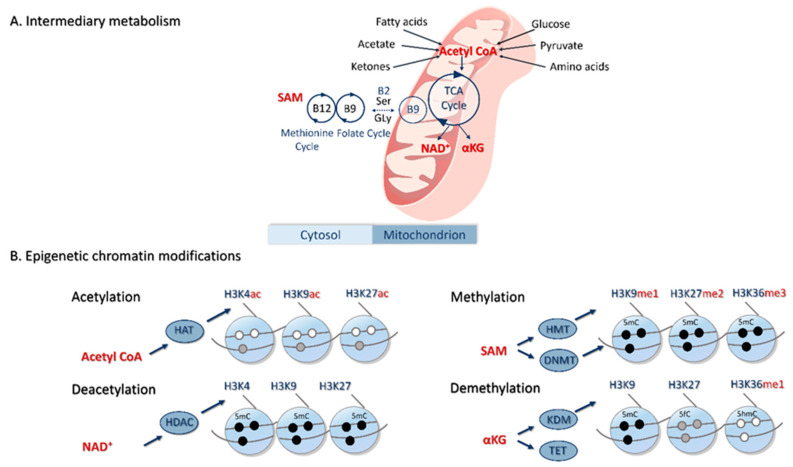
Alterations to intermediary metabolism (**A**) that can result in epigenetic modifications to chromatin (**B**). Intermediary metabolism can be altered directly by dietary composition (during sensitive periods; see Figure 1), and/or by eating disorders and the gut microbiome (see Figure 2). This in turn can alter the availability of various intermediary metabolites that serve as substrates and co-factors for enzymes involved in chromatin modification (Histone acetyltransferases (HAT) and Histone deacetylases (HDAC; e.g., Sirtuins), Histone methyl transferase (HMT) and DNA methyltransferases (DNMTs), Histone (lysine) (KDM) and Ten-Eleven Translocation (TET) demethylases) [80]. Only four of the better-studied intermediary metabolites (i.e., Acetyl CoA, Nicotinamide adenine dinucleotide (NAD^+^), S-adenosylmethionine (SAM) and alpha-ketoglutarate (αKG)) associated with mitochondrial metabolism are presented [81]. Similarly, the best-studied histone acetylation sites are found on various lysine (K) residues on histones H3 and H4, although they also occur on H2A and H2B. Examples of histone (lysine) (mono-[me1], di-[me2] and tri-[me3]) are presented, although arginine methylation should not be discounted [82]. Covalent modifications to DNA include 5-methylcytosine (5mC), 5-formylcytosine (5fC) and 5-hydroxymethylcytosine (5hmC) which are carefully choreographed with histone acetylation/methylation to alter the configuration of chromatin between expressive and repressive states [83]. The generation of small RNAs that can modify chromatin structure and alter transcription is not represented. TCA, tricarboxylic-acid cycle; B2, riboflavin; B9, folate; and B12, cobalamin.

Derangements in 1C metabolism (i.e., linked methionine-folate cycles; Figure 3), which can lead to elevated total homocysteine (tHcy) concentrations in serum and follicular fluid, are implicated in the etiology of PCOS [73,74]. Furthermore, serum tHcy concentrations increase between childhood and adolescence [75], and high concentrations are associated with a number of psychiatric disorders including anxiety and depression [76,77]. In such circumstances, dietary folic acid supplement use can reduce serum tHcy and improve the depressive status of individuals with eating disorders [78]. Similarly, a cocktail of 1C metabolites (including betaine, cobalamin and folate) offered to young women with PCOS was found to reduce fasting serum tHcy and to increase serum AMH concentrations [79]. To date, however, no study has directly assessed the effects of dietary disturbances in 1C metabolism during adolescence on psychological stress, eating disorders and PCOS combined.

## 4. The Prenatal Origin of PCOS

Recent animal and human studies suggest that a steroidal environment and impaired nutrition during in utero development play key roles in the development of PCOS [3]. It appears that AMH acts centrally to exacerbate GnRH- and LH-driven ovarian steroidogenesis, follicular arrest and overproduction of AMH in the ovary later on in life. During pregnancy, this leads to an AMH-driven inhibition of aromatase expression in the placenta which, in turn, leads to an increase in testosterone bioavailability and an overexposure of female offspring to androgens. Indeed, the expression of genes that regulate serotonin and GABA neurotransmitters involved in emotion regulation and anxious behavior was altered in the amygdala of offspring from pregnant rats exposed to high levels of testosterone [84]. There are several animal models that convincingly demonstrate an effect of prenatal androgenization on the etiology of PCOS linked to offspring anxiety and depression [85]. In addition, some epidemiologic studies have shown that adolescents with early features of PCOS were more often born with lower birth weights [86], whilst others found that excess birth weight led to obesity, metabolic syndrome, precocious puberty onset and PCOS during adolescence [87,88].

Collectively, these observations are in line with the developmental origins of the health and disease (DOHaD) paradigm which states that maternal psychological distress and/or poor nutrition during pregnancy can have long-term detrimental effects on offspring health, predisposing them to cardio-vascular and metabolic diseases and other disorders in later life. In women with PCOS (F0), perhaps originating during adolescence, hormonal imbalance during gestation can contribute to an increased risk of their adolescent children (F1) developing a broad spectrum of psychological disorders (including anxiety and eating disorders [89,90]), together with PCOS [86] (Figure 1 and Figure 2). As stated earlier, prenatal exposure to androgens, testosterone or dihydrotestosterone generates the closest PCOS-like phenotype in a variety of animal models [11]. Indeed, it was recently shown that AMH levels in pregnant women with PCOS are around two times higher compared to healthy pregnant controls. Moreover, they were still elevated during the third trimester of pregnancy, a period during which the female fetus is sensitive to androgenization [91].

### 4.1. Anti-Müllerian Hormone

AMH is secreted by primary and growing small antral follicles and inhibits further follicular recruitment from the primordial follicle pool. AMH is also important in attenuating follicular sensitivity to cyclical FSH action, leading to the selection of the dominant follicle [92], and decreasing aromatase activity and the number of luteinizing hormone/choriogonadotropin receptors (LHCGR) in these cells [93]. Mature neurons in the adult brain express high levels of AMH receptors type II (AMHR2) in both sexes. AMHR2 is expressed in a significant subset of hypothalamic GnRH neurons in both mice and humans, and also in different brain areas and cell types involved in the central control of reproduction. These include the organum vasculosum laminae terminalis (OVLT) of the hypothalamus and the median eminence. Within the median eminence, AMHR2 is expressed by endothelial cells, tanycytes and the majority of arcuate nucleus neurons. Fenestrated endothelial cells and tanycytes regulate GnRH secretion by interacting closely with GnRH terminals in the median eminence [94]. Moreover, stimulation of GnRH neurons with AMH (both in vitro and in vivo) leads to increased firing frequency of GnRH neurons, resulting in LH over-secretion and a relative under-excretion of FSH form the pituitary [95].

Recently, it has been shown that AMH is expressed in migratory GnRH neurons in both mice and humans during embryonic development. AMH thus serves as a pro-motility factor for GnRH neurons. Pathohistological analysis of Amhr2-deficient mice showed abnormal development of the peripheral olfactory system and defective embryonic migration of neuroendocrine GnRH cells to the basal forebrain, which results in reduced fertility in adults [96]. Furthermore, studies with pregnant mice found that intrauterine treatment with high concentrations of AMH led to the production of offspring that exhibited typical features of PCOS, such as hyperandrogenism in combination with increased LH pulses. In such circumstances, female offspring had fewer ovulations [97].

Taken together, it appears that AMH acts centrally to exacerbate GnRH- and LH-driven ovarian steroidogenesis, follicular arrest and overproduction of AMH in the ovary later on in life. During pregnancy, this leads to an AMH-driven inhibition of aromatase expression in the placenta which, in turn, leads to an increase in testosterone bioavailability and an overexposure of female offspring to androgens. This is known to be associated with the PCOS phenotype. Indeed, in humans, AMH has been shown to modify the enzymatic activity of steroid hormone synthesis, and women with PCOS have been reported to have reduced placental aromatase activity and increased steroidogenic activity [98]. These data are strongly supported by the group of Giacobini in humans as well as in animal models, indicating a crucial role for AMH in the pathogenesis of PCOS [99,100].

### 4.2. Prenatal Metabolism and Stress

Women with mood, anxiety and eating disorders are at increased risk of pregnancy complications associated with adverse maternal and perinatal outcomes [101,102]. Among these complications is hyperemesis gravidarum leading to dehydration, electrolyte imbalance and malnutrition. Whilst relatively rare (<1% pregnancies), this condition can, together with nausea and vomiting (50–90% of all pregnancies), lead to temporal imbalances in key water-soluble nutrients such as B vitamins (Figure 3), with potential longer-term consequences for fetal development and offspring health including (neuro)endocrine disorders leading to PCOS. Of greater importance is the prevalence of gestational diabetes in women with PCOS which, at around 40%, is 3- to 5-fold higher than that of healthy women [103]. Adipocyte function in adult (~30 years) offspring from such pregnancies is epigenetically modified (discussed later) in such a way as to possibly predispose individuals to metabolic syndrome [104] leading to PCOS. Further, there is now compelling evidence to link exposure to a hyperandrogenic intra-uterine environment with PCOS during offspring adult life [105]. A sequelae to such exposure is adipose tissue dysfunction in adulthood, which is implicated in the pathophysiology of PCOS. It was recently demonstrated, in a prenatally androgenized sheep model of PCOS, that adipogenesis is impaired in subcutaneous tissues during adolescence only to undergo compensatory hypertrophy during adulthood, paralleled with an overexpression of various inflammatory mediators in concert with dyslipidemia indicators of visceral fat accumulation [106]. Finally, thrombophilia and recurrent pregnancy loss are more extreme features of PCOS prevalent across different ethnic populations and highly correlated with genetic variants in specific folate cycle enzymes (Figure 3), plasma concentrations of tHcy, testosterone and measures of insulin resistance and obesity [107,108,109,110].

### 4.3. Prenatal Stress and the Microbiota

Perinatal mood and anxiety disorders (PMAD), arising in part due to fluctuating levels of gonadal steroids, represent a broad category of psychological conditions that typically affect around 10–20% of women. PMAD are known to unfavorably alter the vaginal microbiota [42], which is important given that, in eutherian mammals, microbial symbionts which may be present in utero [111] are known to be transmitted to offspring during transit through the birth canal, and subsequently via breast milk and by maternal contact [112,113]. They are thus subject to modification by factors such as pregnancy-related anxiety, which has been found to unfavorably alter the composition of microbiota isolated from newborn meconium and infant stool [114,115], with potential longer-term implications for cardio-vascular and metabolic health and PCOS.

Extending beyond the mode of delivery (i.e., vaginal delivery vs. caesarian section), at parturition, microbial colonization during the early period of infancy is also influenced by the nature of feeding (i.e., breast vs. formula milk). Whilst data on long-term health implications of such interventions are emerging from studies in both neonatal humans and animals, the focus to date has primarily centered on the development of allergies and metabolic disorders including obesity in offspring [116], with little consideration given to psychiatric conditions and reproductive disorders, such as PCOS, that may also originate during this period.

## 5. Epigenetic Basis of PCOS

An increasing body of data from both human and animal studies has emerged in recent years that describes the epigenetic basis for the developmental origins of PCOS. From epigenome-wide association studies (EWAS) in humans, a series of functional pathways (linked to metabolic and psychiatric comorbidities, as well as autoimmune diseases (e.g., type I diabetes)) have been identified that are consistent across a broad range of cell/tissue types from individuals of diverse geographical origin and ethnic background [117]. These observations indicate common underlying signaling networks involved both in the etiology and heterogeneity of PCOS including pathways linked to mitochondrial metabolism [118]. It is important to note that underlying epigenetic mechanisms extend beyond covalent modifications to histones and DNA depicted in Figure 3 to include interacting long non-coding and microRNAs that are known to influence DNA methylation in PCOS [119]. These are also metabolically regulated [80] and are associated with insulin resistance and lipid disorders in PCOS women [120]. However, evidence for their direct actions in PCOS is limited [121] and this, together with the diverse variety of RNA species and mechanisms of biogenesis, prohibits extensive consideration in this article.

### 5.1. Peripubertal Diet, Epigenetics and PCOS

The methylation status of several growth-related and sex steroid genes (e.g., *IGF2*, *CYP19A1* and *HSD11B2*) is influenced by diet and can affect the timing of puberty onset in adolescent girls [122,123]. A wider role for genomic imprinting in the timing of puberty onset has recently attracted attention given that mutations in at least two imprinted genes (i.e., Makorin Ring Finger Protein 3 (*MKRN3*) and Delta-like noncanonical Notch ligand 1 (*DLK1*)) lead to precocious puberty onset in girls [124]. Mutations in genes encoding kisspeptin (*KISS1*) and its receptor (*KISS1R*) (both activators GnRH secretion) can also advance the onset of puberty. Together with neurokinin B and dynorphin, kisspeptin is produced by a complex of neurons, referred to as KDNy neurons, located in the arcuate nucleus of the hypothalamus and which regulate GnRH secretion. KNDy neurons are subject to epigenetic control and can be modified by a group of energy-sensing proteins called sirtuins, which are tightly coupled to prevailing levels of the metabolic factor NAD^+^ (Figure 3). A recent study with Wister rats demonstrated that the eviction of SIRT1 from hypothalamic Kiss1 neurons created a permissive chromatin state, the timing of which was accelerated by overnutrition, thereby advancing puberty onset [125].

A further study in mice offering a diet deficient in folate, methionine and choline (Figure 3) during their period of adolescence (i.e., 3–6 weeks of age) reported an increase in plasma tHcy concentrations leading to promotor hypermethylation and loss of expression of *Gria1* (encoding Glutamate receptor 1) in the hippocampus, thus impairing memory learning and fear extinction [126]. Further, key regulatory sequences of mitochondrial DNA (mtDNA) were hypermethylated in oocytes from polycystic and hyperhomocysteinemic ovaries of peripubertal pigs, leading to a reduced mtDNA copy number and transcript expression, together with compromised post-fertilization development [127]. Although not specifically measured in these two studies, reported derangements in 1C metabolism would have undoubtedly altered S-adenosylmethionine (SAM)-mediated provision of methyl groups required for these epigenetic modifications (Figure 3) [128].

Collectively, these studies establish the epigenetic basis for the dietary-mediated timing of puberty onset and the neuroendocrine regulation of ovarian function in mammals. However, although proposed as a mechanism underpinning the onset of PCOS in adolescent girls [129], there is currently a lack of direct evidence supporting the epigenetic basis for PCOS inception during puberty in humans, and the effects of potential interacting psychiatric comorbidities remain to be explored.

### 5.2. Prenatal Diet, Epigenetics and PCOS

Our limited understanding of the epigenetic basis for the fetal origins of PCOS is based primarily on work undertaken in animal models, such as rodents, sheep and non-human primates. These studies have assessed the effects of maternal diet/metabolism during pregnancy, modeled the consequences of prenatal androgenization and investigated the outcome of in utero exposure to environmental chemicals [130,131,132].

In the case of prenatal androgenization, treatment exposures have generally been undertaken during early/mid-gestation, reflecting a key stage of androgen-sensitive gonadal development across species [132]. By way of example, prenatal androgenization in rats led to genome-wide alterations in DNA methylation in the ovaries of adult female offspring reflecting a variety of gene networks associated with adult PCOS [133]. Similarly, prenatal androgen exposure in sheep led to upregulation of H3K9me3 (gene repressive), and H3K27ac and H3K9ac (both gene activating) marks in the ovaries of adult offspring, affecting genes involved in steroid biosynthesis and inflammation [134]. In a separate study undertaken in mice, blood concentrations of the methyl donor SAM were reduced in prenatally androgenized offspring in line with attenuated ovarian expression of both Mtr and Bhmt (two 1C cycle enzymes involved in the remethylation of Hcy to methionine (Figure 3)) [135]. It transpires that the majority of genes encoding 1C enzymes are steroid-responsive, accounting for differences in expression/activity during both the estrous and menstrual cycles of mammals, as well as during pregnancy [128]. Significantly, the reported observations of prenatal androgenization in mice are consistent with reduced granulosa cell MTR expression and serum SAM concentrations in hyperandrogenic PCOS women [135].

Evidence from human studies supports the contention that the in utero environment can influence the genesis of PCOS and related comorbidities. Women diagnosed with PCOS can give rise to children that exhibit sex-dependent DNA methylation patterns in regulatory regions of various metabolic and reproductive genes (including adiponectin, AMH and the androgen receptor) associated with this condition [136,137]. In such studies, however, it is difficult to disentangle the effects of maternal genotype. Therefore, analyzing data from a large Swedish nationwide register-based cohort study, and a case–control study from Chile, Risal et al. [138] initially confirmed that daughters of women with PCOS are more likely to be diagnosed with PCOS, and then went on to demonstrate that prenatal androgenization in an inbred strain of mouse led to transgenerational transmission of PCOS up to at least F3 daughters.

## 6. Hypothesis and Conclusions

From this background, we postulate that exposure to a variety of psychological stressors during puberty and adolescence induces chronic psychiatric disorders which, in some individuals, result in repeated episodes of overeating and dieting, collectively contributing to the development of PCOS (Figure 2A). Psychological stressors, such as difficulties at school or at home, low self-esteem or being bullied, are proximal determinants of anxiety, depression and premenstrual dysphoric disorder that are often observed in young women. We propose that psychological distress and associated eating disorders during these periods lead to the epigenetic (Figure 3) dysregulation of (neuro)hormones (e.g., AMH, androgens, estradiol, insulin and ghrelin) involved in the pubertal upregulation of the HPG axis, which contributes to the development of PCOS and related comorbidities in these subjects (Figure 2B).

With its primary focus on puberty and adolescence, this hypothesis should be viewed as an extension, and not a substitute, to current concepts on the developmental origins of PCOS which, to date, have largely centered on endocrine and metabolic disturbances that occur prenatally. Data to support the fetal origins of PCOS are compelling, and the central role of AMH and prenatal androgenization is well established. In women with PCOS (F0; Figure 1), perhaps originating during adolescence, hormonal/metabolic imbalances during gestation can contribute to an increased risk of their adolescent children (F1) developing a broad spectrum of psychological conditions (including anxiety and eating disorders) which, in turn, can enhance their chances of developing PCOS (Figure 2). Establishing timing and causality in our peripubertal model requires prospective cohort studies from infancy. Mechanistic studies should consider the role of the gut microbiota and disturbances to intermediary metabolism, including 1C metabolism, in the epigenetic regulation of (neuro)hormones.

Finally, clinicians should consider the importance of underlying chronic psychological distress and eating disorders in the etiology of PCOS during both pregnancy and puberty. However, this does not preclude clinicians from implementing dietary assessment and evidence-based behavioral interventions as part of preconception and reproductive care (e.g., www.smarterpregnancy.co.uk [139,140,141]). In particular, treatment may be warranted in the case of obesity or severe emotional problems, with cognitive-behavioral therapy, with or without a lifestyle component focused on weight loss, being most commonly recommended [142].

## Abbreviations

1C	one carbon
5mC	include 5-methylcytosine
5fC	5-formylcytosine
5hmC	5-hydroxymethylcytosine
5-HIAA	5-hydroxyindolacetic acid
AG	acylated ghrelin
AMH	anti-Müllerian hormone
AMHR2	anti-Müllerian hormone receptor type II
AN	anorexia nervosa
B12	cobalamin
B2	riboflavin
B9	folate
CYP19A1	cytochrome P450 family 19 subfamily A member 1
DLK1	delta-like noncanonical Notch ligand 1
DNMT’s	DNA methyltransferases
DOHaD	developmental origins of health and disease
EWAS	epigenome-wide association studies
FSH	follicle-stimulating hormone
GABA	γ-amino butyric acid
GLP1	ghrelin and glucagon-like peptide 1
GnRH	gonadotropin-releasing hormone
Gria1	encoding glutamate receptor 1
H3K9ac	histone 3, Lysine 9 acetylation
H3K9me3	histone 3, lysine 9 trimethylation
H3K27ac	histone 3, lysine 27 acetylation
HA	hyperandrogenic
HAT	histone acetyltransferases
HDAC	histone deacetylase
HMT	histone methyltransferase
HPG	hypothalamic-pituitary-gonadal
HSD11B2	hydroxysteroid 11-beta dehydrogenase 2
IGF2	insulin-like growth factor 2
KDM	lysine demethylase
KDNy	kisspeptin, neurokinin B, and dynorphin neurons
KISS1	kisspeptin
KISS1R	kisspeptin receptor
LH	luteinizing hormone
LHCGR	luteinizing hormone/choriogonadotropin receptors
mtDNA	mitochondrial DNA
MTR	methionine synthase
MKRN3	makorin ring finger protein 3
NAD^+^	nicotinamide adenine dinucleotide
OVLT	organum vasculosum laminae terminalis
PCOM	polycystic ovary morphology
PCOS	polycystic ovary syndrome
PMAD	perinatal mood and anxiety disorders
SAM	S-adenosylmethionine
TCA	tricarboxylic-acid cycle
TET	ten-eleven translocation
tHcy	total homocysteine
UAG	unacylated ghrelin
αKG	alpha-ketoglutarate

## References

[1] ESHRE T.T., ASRM-Sponsored PCOS Consensus Workshop Group (2008). Consensus on infertility treatment related to polycystic ovary syndrome. Fertil. Steril..

[2] Blay S.L., Aguiar J.V., Passos I.C. (2016). Polycystic ovary syndrome and mental disorders: A systematic review and exploratory meta-analysis. Neuropsychiatr. Dis. Treat..

[3] Dumesic D.A., Schramm R.D., Abbott D.H. (2005). Early origins of polycystic ovary syndrome. Reprod. Fertil. Dev..

[4] Dunaif A. (2011). Polycystic ovary syndrome in 2011: Genes, aging and sleep apnea in polycystic ovary syndrome. Nat. Rev. Endocrinol..

[5] Azziz R. (2016). Pcos in 2015: New insights into the genetics of polycystic ovary syndrome. Nat. Rev. Endocrinol..

[6] Balen A.H., Morley L.C., Misso M., Franks S., Legro R.S., Wijeyaratne C.N., Stener-Victorin E., Fauser B.C., Norman R.J., Teede H. (2016). The management of anovulatory infertility in women with polycystic ovary syndrome: An analysis of the evidence to support the development of global who guidance. Hum. Reprod. Update.

[7] Teede H., Deeks A., Moran L. (2010). Polycystic ovary syndrome: A complex condition with psychological, reproductive and metabolic manifestations that impacts on health across the lifespan. BMC Med..

[8] ESHRE/ASRM (2004). Rotterdam sponsored pcos consensus workshop group revised 2003 consensus on diagnostic criteria and long-term health risks related to polycystic ovary syndrome. Fertil. Steril..

[9] Witchel S.F., Roumimper H., Oberfield S. (2016). Polycystic ovary syndrome in adolescents. Endocrinol. Metab. Clin. N. Am..

[10] Ibáñez L., Potau N., Francois I., de Zegher F. (1998). Precocious pubarche, hyperinsulinism, and ovarian hyperandrogenism in girls: Relation to reduced fetal growth. J. Clin. Endocrinol. Metab..

[11] Walters K.A., Gilchrist R.B., Ledger W.L., Teede H.J., Handelsman D.J., Campbell R.E. (2018). New perspectives on the pathogenesis of pcos: Neuroendocrine origins. Trends Endocrinol. Metab..

[12] Laven J.S., Mulders A.G., Visser J.A., Themmen A.P., De Jong F.H., Fauser B.C. (2004). Anti-müllerian hormone serum concentrations in normoovulatory and anovulatory women of reproductive age. J. Clin. Endocrinol. Metab..

[13] Steegers E.A., Fauser B.C., Hilders C.G., Jaddoe V.W., Massuger L.F., Schoenmakers S., van der Post J.A. (2019). Textbook of Obstetrics and Gynaecology: A Life Course Approach.

[14] Thannickal A., Brutocao C., Alsawas M., Morrow A., Zaiem F., Murad M.H., Javed Chattha A. (2020). Eating, sleeping and sexual function disorders in women with polycystic ovary syndrome (pcos): A systematic review and meta-analysis. Clin. Endocrinol..

[15] Brutocao C., Zaiem F., Alsawas M., Morrow A.S., Murad M.H., Javed A. (2018). Psychiatric disorders in women with polycystic ovary syndrome: A systematic review and meta-analysis. Endocrine.

[16] Damone A.L., Joham A.E., Loxton D., Earnest A., Teede H.J., Moran L.J. (2019). Depression, anxiety and perceived stress in women with and without pcos: A community-based study. Psychol. Med..

[17] Kerchner A., Lester W., Stuart S.P., Dokras A. (2009). Risk of depression and other mental health disorders in women with polycystic ovary syndrome: A longitudinal study. Fertil. Steril..

[18] Himelein M.J., Thatcher S.S. (2006). Polycystic ovary syndrome and mental health: A review. Obstet. Gynecol. Surv..

[19] Murray K.M., Byrne D.G., Rieger E. (2011). Investigating adolescent stress and body image. J. Adolesc..

[20] Beck A.T. (2008). The evolution of the cognitive model of depression and its neurobiological correlates. Am. J. Psychiatry.

[21] Baker J.H., Girdler S.S., Bulik C.M. (2012). The role of reproductive hormones in the development and maintenance of eating disorders. Expert Rev. Obstet. Gynecol..

[22] Dong Y.-Z., Zhou F.-J., Sun Y.-P. (2017). Psychological stress is related to a decrease of serum anti-müllerian hormone level in infertile women. Reprod. Biol. Endocrinol..

[23] Nestler J.E., Jakubowicz D.J., de Vargas A.F., Brik C., Quintero N., Medina F. (1998). Insulin stimulates testosterone biosynthesis by human thecal cells from women with polycystic ovary syndrome by activating its own receptor and using inositolglycan mediators as the signal transduction system. J. Clin. Endocrinol. Metab..

[24] Algars M., Huang L., Von Holle A.F., Peat C.M., Thornton L.M., Lichtenstein P., Bulik C.M. (2014). Binge eating and menstrual dysfunction. J. Psychosom. Res..

[25] Dallman M.F. (2010). Stress-induced obesity and the emotional nervous system. Trends Endocrinol. Metab..

[26] Escobar-Morreale H., Millán J. (2007). Abdominal adiposity and the polycystic ovary syndrome. Trends Endocrinol. Metab..

[27] Larsen J.K., van Ramshorst B., van Doornen L.J., Geenen R. (2009). Salivary cortisol and binge eating disorder in obese women after surgery for morbid obesity. Int. J. Behav. Med..

[28] Vgontzas A.N., Legro R.S., Bixler E.O., Grayev A., Kales A., Chrousos G.P. (2001). Polycystic ovary syndrome is associated with obstructive sleep apnea and daytime sleepiness: Role of insulin resistance. J. Clin. Endocrinol. Metab..

[29] El-Haschimi K., Pierroz D.D., Hileman S.M., Bjorbaek C., Flier J.S. (2000). Two defects contribute to hypothalamic leptin resistance in mice with diet-induced obesity. J. Clin. Investig..

[30] Jacobs H.S., Conway G.S. (1999). Leptin, polycystic ovaries and polycystic ovary syndrome. Hum. Reprod. Update.

[31] Delhanty P.J., Neggers S.J., van der Lely A.J. (2012). Mechanisms in endocrinology: Ghrelin: The differences between acyl- and des-acyl ghrelin. Eur. J. Endocrinol..

[32] Menzies J.R., Skibicka K.P., Leng G., Dickson S.L. (2013). Ghrelin, reward and motivation. Endocr. Dev..

[33] Perello M., Dickson S.L. (2015). Ghrelin signalling on food reward: A salient link between the gut and the mesolimbic system. J. Neuroendocrinol..

[34] Valdivia S., Cornejo M.P., Reynaldo M., De Francesco P.N., Perello M. (2015). Escalation in high fat intake in a binge eating model differentially engages dopamine neurons of the ventral tegmental area and requires ghrelin signaling. Psychoneuroendocrinology.

[35] King S.J., Rodrigues T., Watts A., Murray E., Wilson A., Abizaid A. (2016). Investigation of a role for ghrelin signaling in binge-like feeding in mice under limited access to high-fat diet. Neuroscience.

[36] Kuppens R.J., Diene G., Bakker N.E., Molinas C., Faye S., Nicolino M., Bernoux D., Delhanty P.J., van der Lely A.J., Allas S. (2015). Elevated ratio of acylated to unacylated ghrelin in children and young adults with prader-willi syndrome. Endocrine.

[37] Delhanty P.J., Neggers S.J., van der Lely A.J. (2013). Des-acyl ghrelin: A metabolically active peptide. Endocr. Dev..

[38] Shi X., Zhang L., Fu S., Li N. (2011). Co-involvement of psychological and neurological abnormalities in infertility with polycystic ovarian syndrome. Arch. Gynecol. Obstet..

[39] Chaudhari N., Dawalbhakta M., Nampoothiri L. (2018). Gnrh dysregulation in polycystic ovarian syndrome (pcos) is a manifestation of an altered neurotransmitter profile. Reprod. Biol. Endocrinol..

[40] Kaye W.H., Frank G.K., Bailer U.F., Henry S.E., Meltzer C.C., Price J.C., Mathis C.A., Wagner A. (2005). Serotonin alterations in anorexia and bulimia nervosa: New insights from imaging studies. Physiol. Behav..

[41] Lam Y.Y., Maguire S., Palacios T., Caterson I.D. (2017). Are the gut bacteria telling us to eat or not to eat? Reviewing the role of gut microbiota in the etiology, disease progression and treatment of eating disorders. Nutrients.

[42] Rackers H.S., Thomas S., Williamson K., Posey R., Kimmel M.C. (2018). Emerging literature in the microbiota-brain axis and perinatal mood and anxiety disorders. Psychoneuroendocrinology.

[43] He F.-F., Li Y.-M. (2020). Role of gut microbiota in the development of insulin resistance and the mechanism underlying polycystic ovary syndrome: A review. J. Ovarian Res..

[44] Qi X., Chuyu Y., Sun L., Xia J., Wu Q., Wang Y., Wang L., Zhang Y., Liang X., Wang L. (2019). Gut microbiota–bile acid–interleukin-22 axis orchestrates polycystic ovary syndrome. Nat. Med..

[45] Zhao X., Jiang Y., Xi H., Chen L., Feng X. (2020). Exploration of the relationship between gut microbiota and polycystic ovary syndrome (pcos): A review. Geburtshilfe Frauenheilkd.

[46] Asarian L., Geary N. (2006). Modulation of appetite by gonadal steroid hormones. Philos. Trans. R. Soc. Lond B Biol. Sci..

[47] Sundblad C., Bergman L., Eriksson E. (1994). High levels of free testosterone in women with bulimia nervosa. Acta Psychiatr. Scand..

[48] Huijgen N.A., Laven J.S., Labee C.T., Louwers Y.V., Willemsen S.P., Steegers-Theunissen R.P. (2015). Are dieting and dietary inadequacy a second hit in the association with polycystic ovary syndrome severity?. PLoS ONE.

[49] Huijgen N.A., Louwers Y.V., Willemsen S.P., de Vries J.H.M., Steegers-Theunissen R.P.M., Laven J.S.E. (2017). Dietary patterns and the phenotype of polycystic ovary syndrome: The chance of ongoing pregnancy. Reprod. Biomed. Online.

[50] Barr S., Hart K., Reeves S., Sharp K., Jeanes Y.M. (2011). Habitual dietary intake, eating pattern and physical activity of women with polycystic ovary syndrome. Eur. J. Clin. Nutr..

[51] Douglas C.C., Gower B.A., Darnell B.E., Ovalle F., Oster R.A., Azziz R. (2006). Role of diet in the treatment of polycystic ovary syndrome. Fertil. Steril..

[52] Moran L.J., Ko H., Misso M., Marsh K., Noakes M., Talbot M., Frearson M., Thondan M., Stepto N., Teede H.J. (2013). Dietary composition in the treatment of polycystic ovary syndrome: A systematic review to inform evidence-based guidelines. Hum. Reprod. Update.

[53] Moran L.J., Grieger J.A., Mishra G.D., Teede H.J. (2015). The association of a mediterranean-style diet pattern with polycystic ovary syndrome status in a community cohort study. Nutrients.

[54] Chiofalo B., Laganà A.S., Palmara V., Granese R., Corrado G., Mancini E., Vitale S.G., Ban Frangež H., Vrtačnik-Bokal E., Triolo O. (2017). Fasting as possible complementary approach for polycystic ovary syndrome: Hope or hype?. Med. Hypotheses.

[55] Facchinetti F., Unfer V., Dewailly D., Kamenov Z.A., Diamanti-Kandarakis E., Laganà A.S., Nestler J.E., Soulage C.O. (2020). Inositols in polycystic ovary syndrome: An overview on the advances. Trends Endocrinol. Metab..

[56] Evans M.C., Anderson G.M. (2017). Neuroendocrine integration of nutritional signals on reproduction. J. Mol. Endocrinol..

[57] Goss A.M., Chandler-Laney P.C., Ovalle F., Goree L.L., Azziz R., Desmond R.A., Wright Bates G., Gower B.A. (2014). Effects of a eucaloric reduced-carbohydrate diet on body composition and fat distribution in women with pcos. Metabolism.

[58] Gower B.A., Chandler-Laney P.C., Ovalle F., Goree L.L., Azziz R., Desmond R.A., Granger W.M., Goss A.M., Bates G.W. (2013). Favourable metabolic effects of a eucaloric lower-carbohydrate diet in women with pcos. Clin. Endocrinol..

[59] Mehrabani H.H., Salehpour S., Amiri Z., Farahani S.J., Meyer B.J., Tahbaz F. (2012). Beneficial effects of a high-protein, low-glycemic-load hypocaloric diet in overweight and obese women with polycystic ovary syndrome: A randomized controlled intervention study. J. Am. Coll. Nutr..

[60] Sorensen L.B., Soe M., Halkier K.H., Stigsby B., Astrup A. (2012). Effects of increased dietary protein-to-carbohydrate ratios in women with polycystic ovary syndrome. Am. J. Clin. Nutr..

[61] Stamets K., Taylor D.S., Kunselman A., Demers L.M., Pelkman C.L., Legro R.S. (2004). A randomized trial of the effects of two types of short-term hypocaloric diets on weight loss in women with polycystic ovary syndrome. Fertil. Steril..

[62] Toscani M.K., Mario F.M., Radavelli-Bagatini S., Wiltgen D., Matos M.C., Spritzer P.M. (2011). Effect of high-protein or normal-protein diet on weight loss, body composition, hormone, and metabolic profile in southern brazilian women with polycystic ovary syndrome: A randomized study. Gynecol. Endocrinol..

[63] Vargas M.L., Almario R.U., Buchan W., Kim K., Karakas S.E. (2011). Metabolic and endocrine effects of long-chain versus essential omega-3 polyunsaturated fatty acids in polycystic ovary syndrome. Metabolism.

[64] Schaumberg K., Welch E., Breithaupt L., Hübel C., Baker J.H., Munn-Chernoff M.A., Yilmaz Z., Ehrlich S., Mustelin L., Ghaderi A. (2017). The science behind the academy for eating disorders’ nine truths about eating disorders. Eur. Eat. Disord. Rev..

[65] Klump K.L., Gobrogge K.L., Perkins P.S., Thorne D., Sisk C.L., Breedlove S.M. (2006). Preliminary evidence that gonadal hormones organize and activate disordered eating. Psychol. Med..

[66] Klump K.L., Keel P.K., Sisk C., Burt S.A. (2010). Preliminary evidence that estradiol moderates genetic influences on disordered eating attitudes and behaviors during puberty. Psychol. Med..

[67] Kaltiala-Heino R., Rimpelä M., Rissanen A., Rantanen P. (2001). Early puberty and early sexual activity are associated with bulimic-type eating pathology in middle adolescence. J. Adolesc. Health.

[68] Kelly Y., Zilanawala A., Sacker A., Hiatt R., Viner R. (2017). Early puberty in 11-year-old girls: Millennium cohort study findings. Arch. Dis. Child..

[69] Lian Q., Mao Y., Luo S., Zhang S., Tu X., Zuo X., Lou C., Zhou W. (2019). Puberty timing associated with obesity and central obesity in chinese han girls. BMC Pediatr..

[70] Nguyen N.T.K., Fan H.Y., Tsai M.C., Tung T.H., Huynh Q.T.V., Huang S.Y., Chen Y.C. (2020). Nutrient intake through childhood and early menarche onset in girls: Systematic review and meta-analysis. Nutrients.

[71] Rothenberg S.S., Beverley R., Barnard E., Baradaran-Shoraka M., Sanfilippo J.S. (2018). Polycystic ovary syndrome in adolescents. Best Pract. Res. Clin. Obstet. Gynaecol..

[72] Mizgier M., Jarząbek-Bielecka G., Opydo-Szymaczek J., Wendland N., Więckowska B., Kędzia W. (2020). Risk factors of overweight and obesity related to diet and disordered eating attitudes in adolescent girls with clinical features of polycystic ovary syndrome. J. Clin. Med..

[73] Eskandari Z., Sadrkhanlou R.-A., Nejati V., Tizro G. (2016). Pcos women show significantly higher homocysteine level, independent to glucose and e2 level. Int. J. Reprod. Biomed..

[74] Meng Y., Chen X., Peng Z., Liu X., Sun Y., Dai S. (2016). Association between high serum homocysteine levels and biochemical characteristics in women with polycystic ovarian syndrome: A systematic review and meta-analysis. PLoS ONE.

[75] Must A., Jacques P.F., Rogers G., Rosenberg I.H., Selhub J. (2003). Serum total homocysteine concentrations in children and adolescents: Results from the third national health and nutrition examination survey (nhanes iii). J. Nutr..

[76] Chung K.H., Chiou H.Y., Chen Y.H. (2017). Associations between serum homocysteine levels and anxiety and depression among children and adolescents in taiwan. Sci. Rep..

[77] Esnafoglu E., Ozturan D.D. (2020). The relationship of severity of depression with homocysteine, folate, vitamin b12, and vitamin d levels in children and adolescents. Child Adolesc. Ment. Health.

[78] Loria-Kohen V., Gómez-Candela C., Palma-Milla S., Amador-Sastre B., Hernanz A., Bermejo L.M. (2013). A pilot study of folic acid supplementation for improving homocysteine levels, cognitive and depressive status in eating disorders. Nutr. Hosp..

[79] Schiuma N., Costantino A., Bartolotti T., Dattilo M., Bini V., Aglietti M.C., Renga M., Favilli A., Falorni A., Gerli S. (2020). Micronutrients in support to the one carbon cycle for the modulation of blood fasting homocysteine in pcos women. J. Endocrinol. Investig..

[80] Sharma U., Rando O.J. (2017). Metabolic inputs into the epigenome. Cell Metab..

[81] Wiese M., Bannister A.J. (2020). Two genomes, one cell: Mitochondrial-nuclear coordination via epigenetic pathways. Mol. Metab..

[82] Bannister A.J., Kouzarides T. (2011). Regulation of chromatin by histone modifications. Cell Res..

[83] Patel D. (2016). A structural perspective on readout of epigenetic histone and DNA methylation marks. Cold Spring Harb. Perspect. Biol..

[84] Hu M., Richard J.E., Maliqueo M., Kokosar M., Fornes R., Benrick A., Jansson T., Ohlsson C., Wu X., Skibicka K.P. (2015). Maternal testosterone exposure increases anxiety-like behavior and impacts the limbic system in the offspring. Proc. Natl. Acad. Sci. USA.

[85] Stener-Victorin E., Padmanabhan V., Walters K.A., Campbell R.E., Benrick A., Giacobini P., Dumesic D.A., Abbott D.H. (2020). Animal models to understand the etiology and pathophysiology of polycystic ovary syndrome. Endocr. Rev..

[86] Sir-Petermann T., Hitchsfeld C., Maliqueo M., Codner E., Echiburú B., Gazitúa R., Recabarren S., Cassorla F. (2005). Birth weight in offspring of mothers with polycystic ovarian syndrome. Hum. Reprod..

[87] Nicandri K.F., Hoeger K. (2012). Diagnosis and treatment of polycystic ovarian syndrome in adolescents. Curr. Opin. Endocrinol. Diabetes Obes..

[88] Qiao Y., Ma J., Wang Y., Li W., Katzmarzyk P.T., Chaput J.P., Fogelholm M., Johnson W.D., Kuriyan R., Kurpad A. (2015). Birth weight and childhood obesity: A 12-country study. Int. J. Obes. Suppl..

[89] Robinson S.L., Ghassabian A., Sundaram R., Trinh M.H., Bell E.M., Mendola P., Yeung E.H. (2020). The associations of maternal polycystic ovary syndrome and hirsutism with behavioral problems in offspring. Fertil. Steril..

[90] Chen X., Kong L., Piltonen T.T., Gissler M., Lavebratt C. (2020). Association of polycystic ovary syndrome or anovulatory infertility with offspring psychiatric and mild neurodevelopmental disorders: A finnish population-based cohort study. Hum. Reprod..

[91] Piltonen T.T., Giacobini P., Edvinsson Å., Hustad S., Lager S., Morin-Papunen L., Tapanainen J.S., Sundström-Poromaa I., Arffman R.K. (2019). Circulating antimüllerian hormone and steroid hormone levels remain high in pregnant women with polycystic ovary syndrome at term. Fertil. Steril..

[92] Durlinger A.L., Gruijters M.J., Kramer P., Karels B., Kumar T.R., Matzuk M.M., Rose U.M., de Jong F.H., Uilenbroek J.T., Grootegoed J.A. (2001). Anti-müllerian hormone attenuates the effects of fsh on follicle development in the mouse ovary. Endocrinology.

[93] Dumesic D.A., Lesnick T.G., Stassart J.P., Ball G.D., Wong A., Abbott D.H. (2009). Intrafollicular antimüllerian hormone levels predict follicle responsiveness to follicle-stimulating hormone (fsh) in normoandrogenic ovulatory women undergoing gonadotropin releasing-hormone analog/recombinant human fsh therapy for in vitro fertilization and embryo transfer. Fertil. Steril..

[94] Prevot V., Dehouck B., Sharif A., Ciofi P., Giacobini P., Clasadonte J. (2018). The versatile tanycyte: A hypothalamic integrator of reproduction and energy metabolism. Endocr. Rev..

[95] Cimino I., Casoni F., Liu X., Messina A., Parkash J., Jamin S.P., Catteau-Jonard S., Collier F., Baroncini M., Dewailly D. (2016). Novel role for anti-müllerian hormone in the regulation of gnrh neuron excitability and hormone secretion. Nat. Commun..

[96] Malone S.A., Papadakis G.E., Messina A., Mimouni N.E.H., Trova S., Imbernon M., Allet C., Cimino I., Acierno J., Cassatella D. (2019). Defective amh signaling disrupts gnrh neuron development and function and contributes to hypogonadotropic hypogonadism. eLife.

[97] Tata B., Mimouni N.E.H., Barbotin A.L., Malone S.A., Loyens A., Pigny P., Dewailly D., Catteau-Jonard S., Sundström-Poromaa I., Piltonen T.T. (2018). Elevated prenatal anti-müllerian hormone reprograms the fetus and induces polycystic ovary syndrome in adulthood. Nat. Med..

[98] Cook C.L., Siow Y., Brenner A.G., Fallat M.E. (2002). Relationship between serum müllerian-inhibiting substance and other reproductive hormones in untreated women with polycystic ovary syndrome and normal women. Fertil. Steril..

[99] Barbotin A.L., Peigné M., Malone S.A., Giacobini P. (2019). Emerging roles of anti-müllerian hormone in hypothalamic-pituitary function. Neuroendocrinology.

[100] Silva M.S.B., Giacobini P. (2020). New insights into anti-müllerian hormone role in the hypothalamic-pituitary-gonadal axis and neuroendocrine development. Cell Mol. Life Sci..

[101] Alder J., Fink N., Bitzer J., Hösli I., Holzgreve W. (2007). Depression and anxiety during pregnancy: A risk factor for obstetric, fetal and neonatal outcome? A critical review of the literature. J. Matern Fetal Neonatal Med..

[102] Hoirisch-Clapauch S., Brenner B., Nardi A.E. (2015). Adverse obstetric and neonatal outcomes in women with mental disorders. Thromb. Res..

[103] Dumesic D.A., Hoyos L.R., Chazenbalk G.D., Naik R., Padmanabhan V., Abbott D.H. (2020). Mechanisms of intergenerational transmission of polycystic ovary syndrome. Reproduction.

[104] Hansen N.S., Strasko K.S., Hjort L., Kelstrup L., Houshmand-Øregaard A., Schrölkamp M., Schultz H.S., Scheele C., Pedersen B.K., Ling C. (2017). Fetal hyperglycemia changes human preadipocyte function in adult life. J. Clin. Endocrinol. Metab..

[105] Filippou P., Homburg R. (2017). Is foetal hyperexposure to androgens a cause of pcos?. Hum. Reprod. Update.

[106] Siemienowicz K.J., Coukan F., Franks S., Rae M.T., Duncan W.C. (2020). Aberrant subcutaneous adipogenesis precedes adult metabolic dysfunction in an ovine model of polycystic ovary syndrome (pcos). Mol. Cell. Endocrinol..

[107] Chakraborty P., Goswami S.K., Rajani S., Sharma S., Kabir S.N., Chakravarty B., Jana K. (2013). Recurrent pregnancy loss in polycystic ovary syndrome: Role of hyperhomocysteinemia and insulin resistance. PLoS ONE.

[108] Kazerooni T., Ghaffarpasand F., Asadi N., Dehkhoda Z., Dehghankhalili M., Kazerooni Y. (2013). Correlation between thrombophilia and recurrent pregnancy loss in patients with polycystic ovary syndrome: A comparative study. J. Chin. Med. Assoc..

[109] Szafarowska M., Segiet A., Jerzak M.M. (2016). Methylenotetrahydrololate reductase a1298c and c677t polymorphisms and adverse pregnancy outcome in women with pcos. Neuro Endocrinol. Lett..

[110] Chang C.L., Huang S.Y., Hsu Y.C., Chin T.H., Soong Y.K. (2019). The serum level of irisin, but not asprosin, is abnormal in polycystic ovary syndrome patients. Sci. Rep..

[111] Mesa M.D., Loureiro B., Iglesia I., Fernandez Gonzalez S., Llurba Olivé E., García Algar O., Solana M.J., Cabero Perez M.J., Sainz T., Martinez L. (2020). The evolving microbiome from pregnancy to early infancy: A comprehensive review. Nutrients.

[112] Kaplan J.L., Shi H.N., Walker W.A. (2011). The role of microbes in developmental immunologic programming. Pediatr. Res..

[113] Kau A.L., Ahern P.P., Griffin N.W., Goodman A.L., Gordon J.I. (2011). Human nutrition, the gut microbiome and the immune system. Nature.

[114] Zijlmans M.A., Korpela K., Riksen-Walraven J.M., de Vos W.M., de Weerth C. (2015). Maternal prenatal stress is associated with the infant intestinal microbiota. Psychoneuroendocrinology.

[115] Hu J., Ly J., Zhang W., Huang Y., Glover V., Peter I., Hurd Y.L., Nomura Y. (2019). Microbiota of newborn meconium is associated with maternal anxiety experienced during pregnancy. Dev. Psychobiol..

[116] Milani C., Duranti S., Bottacini F., Casey E., Turroni F., Mahony J., Belzer C., Delgado Palacio S., Arboleya Montes S., Mancabelli L. (2017). The first microbial colonizers of the human gut: Composition, activities, and health implications of the infant gut microbiota. Microbiol. Mol. Biol. Rev..

[117] Tan Q. (2020). Deciphering the DNA methylome of polycystic ovary syndrome. Mol. Diagn. Ther..

[118] Shukla P., Mukherjee S. (2020). Mitochondrial dysfunction: An emerging link in the pathophysiology of polycystic ovary syndrome. Mitochondrion.

[119] Mao Z., Li T., Zhao H., Qin Y., Wang X., Kang Y. (2020). Identification of epigenetic interactions between microrna and DNA methylation associated with polycystic ovarian syndrome. J. Hum. Genet..

[120] Abdalla M.A., Deshmukh H., Atkin S., Sathyapalan T. (2020). A review of therapeutic options for managing the metabolic aspects of polycystic ovary syndrome. Ther. Adv. Endocrinol. Metab..

[121] Chen B., Xu P., Wang J., Zhang C. (2019). The role of mirna in polycystic ovary syndrome (pcos). Gene.

[122] Stueve T.R., Wolff M.S., Pajak A., Teitelbaum S.L., Chen J. (2014). Cyp19a1 promoter methylation in saliva associated with milestones of pubertal timing in urban girls. BMC Pediatr..

[123] Wu Y., Peterson K.E., Sánchez B.N., Dolinoy D.C., Mercado-Garcia A., Téllez-Rojo M.M., Goodrich J.M. (2018). Association of blood leukocyte DNA methylation at line-1 and growth-related candidate genes with pubertal onset and progression. Epigenetics.

[124] Roberts S.A., Kaiser U.B. (2020). Genetics in endocrinology: Genetic etiologies of central precocious puberty and the role of imprinted genes. Eur. J. Endocrinol..

[125] Vazquez A., Sanchez-Rodriguez E., Vargas F., Montoro-Molina S., Romero M., Espejo-Calvo J.A., Vilchez P., Jaramillo S., Olmo-García L., Carrasco-Pancorbo A. (2019). Cardioprotective effect of a virgin olive oil enriched with bioactive compounds in spontaneously hypertensive rats. Nutrients.

[126] Tomizawa H., Matsuzawa D., Ishii D., Matsuda S., Kawai K., Mashimo Y., Sutoh C., Shimizu E. (2015). Methyl-donor deficiency in adolescence affects memory and epigenetic status in the mouse hippocampus. Genes Brain Behav..

[127] Jia L., Li J., He B., Jia Y., Niu Y., Wang C., Zhao R. (2016). Abnormally activated one-carbon metabolic pathway is associated with mtdna hypermethylation and mitochondrial malfunction in the oocytes of polycystic gilt ovaries. Sci. Rep..

[128] Clare C.E., Brassington A.H., Kwong W.Y., Sinclair K.D. (2019). One-carbon metabolism: Linking nutritional biochemistry to epigenetic programming of long-term development. Annu. Rev. Anim. Biosci..

[129] Ibáñez L., de Zegher F. (2020). Polycystic ovary syndrome in adolescent girls. Pediatr. Obes..

[130] Xu N., Kwon S., Abbott D.H., Geller D.H., Dumesic D.A., Azziz R., Guo X., Goodarzi M.O. (2011). Epigenetic mechanism underlying the development of polycystic ovary syndrome (pcos)-like phenotypes in prenatally androgenized rhesus monkeys. PLoS ONE.

[131] Nilsson E.E., Sadler-Riggleman I., Skinner M.K. (2018). Environmentally induced epigenetic transgenerational inheritance of disease. Environ. Epigenet..

[132] Abbott D.H., Dumesic D.A., Levine J.E. (2019). Hyperandrogenic origins of polycystic ovary syndrome—Implications for pathophysiology and therapy. Expert Rev. Endocrinol. Metab..

[133] Zhang D., Cong J., Shen H., Wu Q., Wu X. (2014). Genome-wide identification of aberrantly methylated promoters in ovarian tissue of prenatally androgenized rats. Fertil. Steril..

[134] Sinha N., Roy S., Huang B., Wang J., Padmanabhan V., Sen A. (2020). Developmental programming: Prenatal testosterone-induced epigenetic modulation and its effect on gene expression in sheep ovary†. Biol. Reprod..

[135] Lei L., Ding L., Su J., Liu M., Shi Q., Zhou J., Sun H., Yan G. (2017). Attenuated expression of mtr in both prenatally androgenized mice and women with the hyperandrogenic phenotype of pcos. PLoS ONE.

[136] Lambertini L., Saul S.R., Copperman A.B., Hammerstad S.S., Yi Z., Zhang W., Tomer Y., Kase N. (2017). Intrauterine reprogramming of the polycystic ovary syndrome: Evidence from a pilot study of cord blood global methylation analysis. Front. Endocrinol..

[137] Echiburú B., Milagro F., Crisosto N., Pérez-Bravo F., Flores C., Arpón A., Salas-Pérez F., Recabarren S.E., Sir-Petermann T., Maliqueo M. (2020). DNA methylation in promoter regions of genes involved in the reproductive and metabolic function of children born to women with pcos. Epigenetics.

[138] Risal S., Pei Y., Lu H., Manti M., Fornes R., Pui H., Zhao Z., Massart J., Ohlsson C., Lindgren E. (2019). Prenatal androgen exposure and transgenerational susceptibility to polycystic ovary syndrome. Nat. Med..

[139] Oostingh E.C., Koster M.P.H., van Dijk M.R., Willemsen S.P., Broekmans F.J.M., Hoek A., Goddijn M., Klijn N.F., van Santbrink E.J.P., Steegers E.A.P. (2020). First effective mhealth nutrition and lifestyle coaching program for subfertile couples undergoing in vitro fertilization treatment: A single-blinded multicenter randomized controlled trial. Fertil. Steril..

[140] Oostingh E.C., Hall J., Koster M.P.H., Grace B., Jauniaux E., Steegers-Theunissen R.P.M. (2019). The impact of maternal lifestyle factors on periconception outcomes: A systematic review of observational studies. Reprod. Biomed. Online.

[141] Van der Windt M., van der Kleij R.M., Snoek K.M., Willemsen S.P., Dykgraaf R.H.M., Laven J.S.E., Schoenmakers S., Steegers-Theunissen R.P.M. (2020). Impact of a blended periconception lifestyle care approach on lifestyle behaviors: Before-and-after study. J. Med. Internet Res..

[142] Lee I., Cooney L.G., Saini S., Smith M.E., Sammel M.D., Allison K.C., Dokras A. (2017). Increased risk of disordered eating in polycystic ovary syndrome. Fertil. Steril..

